# Nurses’ Experience Using Telehealth in the Follow-Up Care of Patients with Inflammatory Bowel Disease—A Scoping Review

**DOI:** 10.3390/nursrep16010011

**Published:** 2025-12-29

**Authors:** Nanda Kristin Sæterøy-Hansen, Marit Hegg Reime

**Affiliations:** 1 Medical Outpatient Clinic, Ålesund Hospital, Åsehaugen 5, 6017 Ålesund, Norway; nanda.kristin.seteroy@helse-mr.no; 2 Department of Health and Caring Sciences, Faculty of Health and Social Sciences, Western Norway University of Applied Sciences, Inndalsveien 28, 5020 Bergen, Norway; 3 Department of Postgraduate Studies, Lovisenberg Diaconal University College, Lovisenberggata 15B, 0456 Oslo, Norway

**Keywords:** digital monitoring, inflammatory bowel disease, nurse experience, remote follow-up, scoping review, telehealth

## Abstract

**Background:** Due to the lack of curative treatments for inflammatory bowel disease (IBD), patients need lifelong follow-up care. Telehealth offers a valuable solution to balance routine visits with necessary monitoring. **Objectives:** To map what is known about the benefits and barriers encountered by nurses in their use of telehealth for the follow-up care of patients with IBD. **Methods:** Following the methodology from the Joanna Briggs Institute, we conducted a scoping review across four electronic databases from June 2024 to September 2025. Key search terms included “inflammatory bowel disease,” “nurse experience,” and “telehealth.” A content analysis was employed to summarize the key findings. **Results:** We screened 1551 records, ultimately including four original research articles from four countries. Benefits identified were as follows: (1) the vital contributions of IBD telenursing in empowering patients by bridging health literacy and self-care skills; (2) optimal use of staffing time supports patient-centred care; and (3) ease of use. Barriers included the following: (1) increased workload and task imbalances; (2) the need for customized interventions; (3) technical issues and concerns regarding the security of digital systems; (4) telehealth as a supplementary option or a standard procedure; and (5) concerns related to the patient–nurse relationship. **Conclusions:** Nurses view telehealth as a promising approach that enhances patients’ health literacy and self-care skills and improves patient outcomes through effective monitoring. To fully realize telehealth’s potential, implementing strategies like triage protocols, algorithmic alerts, electronic health record integration, and comprehensive nurse training to enhance patient care and engagement may be beneficial. This scoping review highlights the need for more research on nurses’ experiences with telehealth in IBD due to limited publications.

## 1. Introduction

Digital health technologies like smartphones, tablets, and Web platforms are rapidly changing the practice of medicine and redefining approaches to healthcare services [1]. Inflammatory bowel disease (IBD), encompassing Crohn’s disease (CD) and ulcerative colitis (UC), is a lifelong chronic condition that is well situated for technological interventions due to its relapsing and remitting nature [1]. IBD has become a global disease in recent decades, with a prevalence of over five million people worldwide [2], and is most prevalent north of the equator [3].

IBD is influenced by environmental and genetic factors, and it is hypothesized that an incorrect immune response occurs against the intestinal microbiota in genetically predisposed individuals [4]. The disease can affect all age groups and is equally distributed between the sexes, but is most frequently seen in young people aged 15–35 years [3]. As UC affects the colon mucosa and may cause perforation, CD affects the entire intestinal system and attacks all layers of the intestinal wall, which may cause stenoses and fistulas [4]. Extraintestinal manifestations, like skin conditions, joint inflammation, primary sclerosing cholangitis, eye inflammation, osteoporosis, and anaemia, may also occur in IBD [4].

Due to the lack of curative treatment for IBD and the unpredictable course of the disease, there is a need for lifelong treatment and follow-up care, through monitoring of symptoms both clinically and endoscopically to prevent progression of the disease [5,6]. Most patients receive outpatient follow-up care and are seen for consultations as needed [6], with clinical evaluations, biomarker monitoring, e.g., faecal calprotectin and C-reactive protein (CRP), and cross-sectional imaging [7]. Patients with IBD are best managed by a dedicated IBD service and a multidisciplinary team, including gastroenterologists, nurses, colorectal surgeons, dieticians, psychologists, pharmacists, radiologists, and pathologists [8]. The IBD nurses play a vital role in supporting clinical care and perform tasks as outlined in the European Crohn’s and Colitis nursing guidelines (N-ECCO) [7,8]. These tasks include providing patient education, counselling, monitoring, and offering both physical and emotional support. Additionally, nurses facilitate patients’ access to healthcare services, including support via telephone and e-mail [7], and the value of IBD nurses providing clinical care has been shown to be cost-effective and contribute to reduced healthcare costs [2,8,9,10,11].

### 1.1. Telehealth

Telehealth has expanded in response to the COVID-19 pandemic [12,13], switching from in-person healthcare delivery to either solely telehealth or hybrid models of care [12]. The decreasing length of hospital stays and increasing use of outpatient care necessitate adoption of new tools for providing nursing care [14]. Additionally, the growing proportion of individuals with chronic and complex health challenges has increased pressure on healthcare delivery systems, addressing a need to make patients active participants in the care they receive [15].

Telehealth is defined as “the provision of healthcare remotely by means of a variety of telecommunication tools” [16,17] and encompasses a variety of strategies, including home monitoring, virtual consultations, forward triage, in-hospital telemedicine, and telerehabilitation [15]. Telehealth has also been defined as an alternative for in-person consultations using video conferencing, internet, and telephone [12]. These modalities can be effectively employed in synchronous and asynchronous formats, and they may also incorporate remote patient monitoring [18]. Synchronous technologies involve real-time communication with patients through methods such as telephone calls or audio-video conferencing, utilizing devices like computers, smartphones, or tablets. In contrast, asynchronous modalities enable the storage of patient-reported data, allowing for analysis and addressing at a later time. Patient-Reported Outcomes (PROs) refer to the subjective reports provided by patients concerning their health status, quality of life, or functional capabilities in relation to healthcare or treatment interventions, without interpretation from clinicians or others [18]. The patient portal serves as a prime example of asynchronous technology, enabling patients to access their health information and communicate with healthcare providers at their convenience. Additionally, remote patient monitoring exemplifies this approach by transmitting data from devices that measure various patient parameters directly to healthcare providers [14]. Hybrid care models combine synchronous in-person or telehealth appointments with the use of asynchronous digital tools [19]. Asynchronous telehealth has become the most prominent method for supporting patients with IBD, with 25% receiving asynchronous follow-up care in 2020 [18]. Prior to the COVID-19 pandemic, 90% of patients with IBD relied solely on in-person follow-up consultations [20]. Interestingly, a significant number of practitioners—68%—reported that they had not conducted any video consultations prior to the pandemic [20]. This landscape shifted dramatically with the onset of the pandemic, which led to a remarkable 154% increase in telehealth usage [20].

### 1.2. Research on Telehealth

Several studies have indicated that digital follow-up maintains the same levels of quality of life, medication adherence, and patient satisfaction as in-person consultations, without leading to an increase in unplanned hospitalizations [10,21]. However, healthcare providers must take individual needs and preferences into account to determine the most appropriate mode of delivering care [9]. The implementation of telehealth applications may significantly reshape nurses’ daily practices, as well as influence the nurse–patient relationship [14]. The telehealth environment differs markedly from in-person interactions, as face-to-face encounters enable both the provider and the patient to observe one another and the surrounding environment clearly, allowing nuanced responses to both verbal and nonverbal cues [22].

Research highlights several benefits of telehealth, such as increased patient engagement and improved health outcomes by empowering patients with chronic conditions, eliminating geographical barriers, reducing healthcare costs, and helping maintain the relationship between healthcare providers and patients, thereby enhancing patient-centred care [12,13,14,15,17]. Patient-centred care is defined as a holistic approach taken by healthcare professionals, which begins with an understanding of the patient as a unique individual before arriving at a diagnosis [23]. This approach involves exploring the patient’s reasons for their visit, understanding their context, establishing a shared understanding of the problem, and enhancing prevention and health promotion measures, as well as the relationship between the patient and healthcare professionals.

Additionally, healthcare personnel from various studies have reported that telehealth is easy to learn and use, and it is generally not perceived as burdensome, time-consuming, or onerous [17]. Barriers to telehealth implementation primarily stem from issues related to reimbursement, licensing laws, practitioner-related regulations, confidentiality, security, and technical challenges [12,14,15,24]. Additionally, acceptance among healthcare personnel has also been identified as a barrier to successful telehealth adoption [17]. Nevertheless, research focusing on nurses’ experiences with telehealth in the context of caring for patients with IBD is limited [25]. Consequently, this scoping review aimed to map published studies on this topic, as scoping reviews are specifically designed to explore the breadth of research related to a particular issue. The specific research question was as follows: What is known from the research literature about the benefits and barriers encountered by nurses in their use of telehealth for the follow-up care of patients with IBD?

## 2. Materials and Methods

Scoping reviews are suitable when one wishes to assess knowledge in an emerging field [26]. This scoping review was conducted according to the Joanna Briggs Institute’s (JBI) methodology for scoping reviews [26]. It follows Arksey and O’Malley [27] methodological guidelines, later refined by Levac et al. [28], Tricco et al. [29], Peters et al. [26] and Pollock et al. [30]. The nine steps involved in conducting a scoping review consist of (1) define the review question, (2) develop the inclusion and exclusion criteria, (3) describe the planned approach to evidence searching, data extraction and presentation of the evidence, (4) search for the evidence, (5) select the evidence, (6) extract the evidence, (7) analyse the evidence, (8) present the results, and (9) summarize the evidence in relation to the purpose of the review [26].

A protocol was developed by using the template from JBI and included pre-defined objectives, methods, and reporting of the review [26]. The protocol was not registered in a database. The Preferred Reporting Items for Systematic reviews and Meta-Analyses extension for Scoping Reviews (PRISMA-ScR) checklist has been used throughout the study to ensure transparency and alignment with JBI guidelines [29] (Supplement S1).

### 2.1. Defining the Review Question

The review question was developed using the Population, Concept and Context (PCC) framework [26]. The population comprised patients with IBD, the concept focused on nurses’ experiences, and the context pertained to telehealth (Table 1).

### 2.2. Developing the Inclusion and Exclusion Criteria

The PCC framework was used to develop the eligibility criteria and make them transparent and in accordance with the research question [26,30]. Eligibility criteria are shown in Table 2, and no year limit for the literature search was applied allowing all research conducted on this topic to be included.

### 2.3. Search Strategy

We conducted a background search on the 6th of March 2024 in the databases Epistemonikos and Cochrane Library with the words “IBD and nursing and digital follow-up” to determine if reviews had already been conducted on this topic, only finding reviews describing patients’ experiences. Likewise, a free text search in Google Scholar did not reveal reviews about IBD nurses’ experiences with telehealth. In addition, an initial search was performed in CINAHL and MEDLINE with a librarian to find relevant index terms and text words to prepare the systematic search strategy, in line with recommendations from the JBI manual [26].

The complete search strategy was peer-reviewed by a senior librarian using the PRESS form (Peer Review of Electronic Search Strategies), recommended as a structured tool for conducting comprehensive searches and minimizing errors in the literature search strategies [31]. Several proximity operators and truncations were then added. A computerized literature search was conducted in CINAHL Complete, MEDLINE Complete, Embase, and Web of Science from 18 June 2024 to 3 July 2024. This search was updated on 24 November 2024, 21 April 2025, and 15 September 2025. A comprehensive electronic search strategy, including keywords and index terms, is detailed in Supplement S2, along with the search history from CINAHL provided in Supplement S3 [29]. Additionally, Table 3 presents the specific search strategy employed in CINAHL.

### 2.4. Screening and Selecting the Sources of Evidence

Studies were exported to EndNote 21 (n = 2250) and then imported to Covidence 2025 [32], a systematic review software tool for archiving, screening, and performing data extraction of articles, allowing collaboration among researchers for blinded screening. Covidence automatically removed duplicates (n = 806); however, an additional 24 duplicates required manual removal (n = 24).

To ensure accuracy and completeness, title and abstract screening was conducted in a blinded and independent manner by two researchers [30]. The eligibility criteria were piloted on the first 25 articles, and inconsistencies were resolved along the way in “conflict meetings” [26,33]. No modifications were made to the inclusion and exclusion criteria following this pilot screening. Subsequently, the remaining articles were screened using the same method, as a 75% or greater agreement was achieved [26].

All full-text studies (n = 28) underwent independent reviews by two researchers, and a pilot test was performed on a selection of five studies to evaluate the eligibility criteria. Any discrepancies identified during this initial review were resolved in “conflict meetings” before proceeding with the independent screening of the remaining articles. Of the 28 studies reviewed, 24 were excluded based on the reasons listed in Supplement S4. Additionally, citation searches (n = 131) were conducted using the reference lists of the four included studies [26].

### 2.5. Data Extraction

The data extraction form was prepared a priori [30]. A blinded pilot test was performed by the authors on two of the articles and then adjusted after a review meeting to ensure applicability to the research question. Thereafter, the form was transferred to Covidence, and the remaining data extraction was carried out there before it was transferred to Microsoft Word (MSO version 2503) to better present the findings (Table 4).

### 2.6. Analysing the Evidence and Summarizing the Findings

To summarize the findings and answer the research question, we employed descriptive trends and inductive content analysis, as inspired by Aveyard [38]. This process involved categorizing the data and narratively describing the categories in accordance with the principles of scoping reviews [26]. The papers were read multiple times to identify patterns, similarities, and differences. The findings were illustrated in a rose diagram, where the size of the blocks reflects the number of studies associated with each sub-category under the main categories. Since the purpose of a scoping review is to map and systematically organize the available research material, regardless of quality, this review follows the PRISMA-ScR guidelines [26].

## 3. Results

### 3.1. Selection of Sources of Evidence

**Figure 1:**
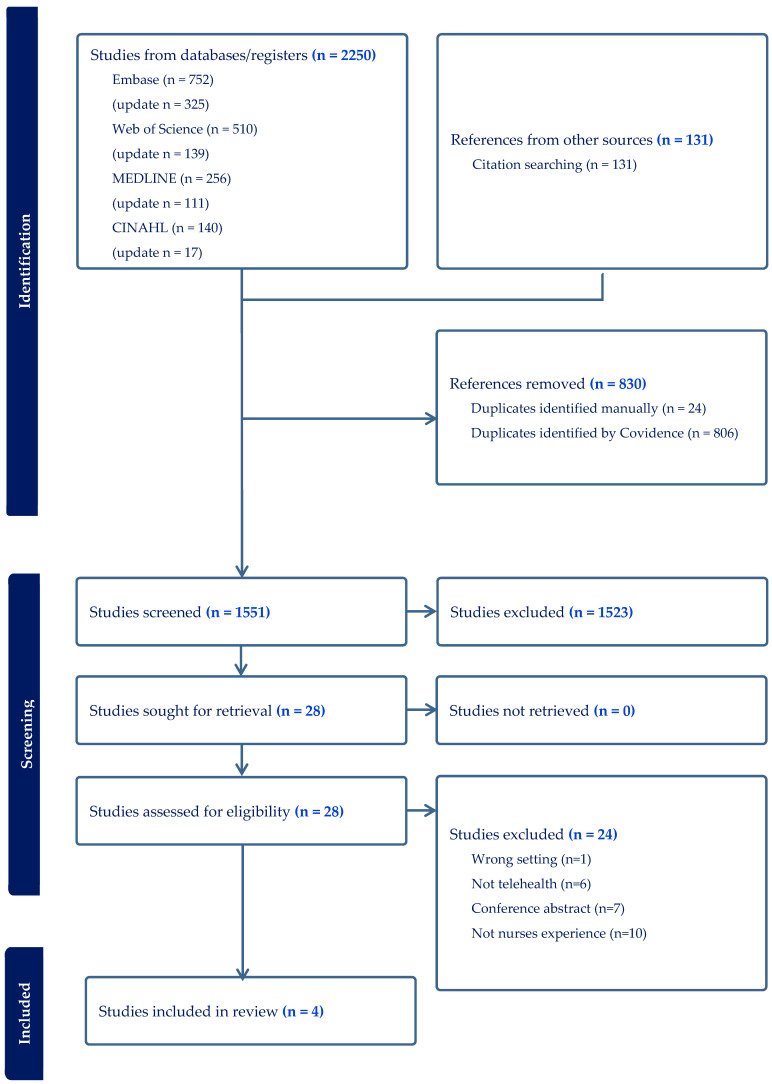
PRISMA-ScR flow diagram.

A total of 2250 records were identified from the database searches, of which 830 were duplicates. This resulted in 1551 titles and abstracts being screened, of which 131 were from articles identified through citation searches. In total, 28 full-text articles were assessed for eligibility, and four of these met the inclusion criteria. The selection process is summarized in a PRISMA-ScR flow diagram (Figure 1) [29].

### 3.2. Characteristics of the Included Studies

The included articles were published between 2016 and 2024 and originated from four different countries: Canada [35], Denmark [34], the United Kingdom [37], and the United States of America [36]. They utilized a variety of methodological approaches, including cross-sectional studies [35], action research [34], case studies [37], and qualitative studies [36]. Data collection methods consisted of individual face-to-face interviews [34,36,37], individual telephone interviews [37], and surveys [35]. The analysis techniques employed included thematic analysis [34,37], rapid qualitative analysis [36], and various statistical analyses [35]. The sample size ranged from two to twenty-one IBD nurse professionals.

The nursing professionals involved in the studies comprised registered nurses [34,35], nurse specialists [37], and nurses working as part of a multidisciplinary team [36]. The digital tools and platforms utilized included digital patient-reported outcome (PRO) platforms (e.g., Ambu-Flex, CAPTURE-IBD) [34,36], patient-held personal health records (e.g., PKB) [37], and email communication [35]. The context of telehealth was characterized as asynchronous [36,37] and hybrid [34,35].

### 3.3. Nurses’ Experience on Performing Telehealth

**Figure 2:**
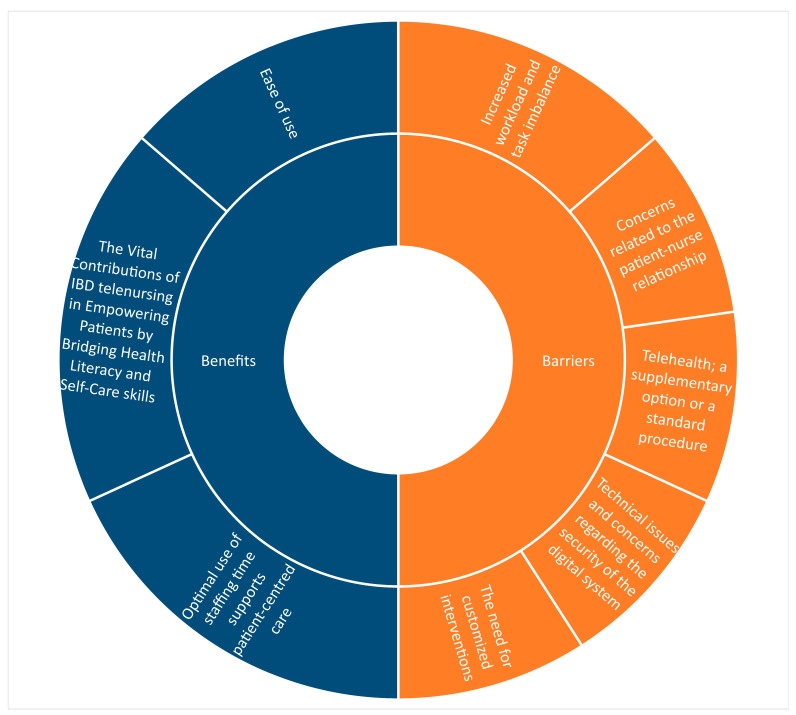
Rose diagram illustrating the benefits and barriers of performing telehealth.

In line with the aim of the study, to identify benefits and barriers in nurses’ experience performing telehealth, we revealed three subcategories capturing benefits, and five sub-categories addressing barriers (Figure 2).

#### 3.3.1. Benefits

- The Vital Contributions of IBD telenursing in Empowering Patients by Bridging Health Literacy and Self-Care skills

IBD nurses believed that digital PROs supported patient education and health literacy skills by improving patients’ disease knowledge and symptom reflections by regularly answering the same PRO questions, revealing symptom variations [34]. Proactive nurse support and access to information empowered patients’ self-care skills and confidence in coping [36,37]. In addition, PRO interventions may connect patients to mental health resources, particularly beneficial for newly diagnosed individuals navigating their healthcare [36], as mental health often remains poorly integrated into IBD care, with around half of IBD patients experiencing comorbid symptoms of depression, anxiety, and stigma [36]. Nurses believed that the telehealth system increased healthcare accessibility [34,35,37] and continuity of care [35,37], particularly for stable IBD patients, who tend to engage with the system more often when feeling unwell and less often when feeling better [37].

IBD nurses experienced playing a crucial role in managing patients’ flare-ups, addressing medical queries, reviewing test results, and delivering care, thereby reducing clinical visits and calls to gastroenterologists [35]. Therefore, IBD nurses should possess skills in effective triage, decision-making, and organize diagnostic tests as part of the follow-up care, as well as participate in advice line training programs to promote patients’ self-care skills [35].

- Optimal use of staffing time supports patient-centred care

Digital consultations reduced waiting times for acute patients by eliminating unnecessary in-person appointments for healthy individuals, allowing nurses to focus on urgent care [34,35,37]. Nurses believed that digital consultations enhanced two-way communication, keeping lines open even when patients were not fully “discharged”, enabling more effective management of stable patients [34,36,37].

IBD nurses also experienced that consultations became more patient-controlled, allowing patients to initiate contact when experiencing increased symptoms and were in need of a follow-up plan [34]. Nurses appreciated the notifications of patient symptoms, aiding in timely monitoring and evaluations and physician alerts [34,35,36,37]. The PKB system employed a “traffic light” code for symptom tracking [35], scoring patients based on the severity of symptoms, enabling early intervention from nurses helping to avoid hospitalizations. IBD nurses handled 61% of patients’ inquiries independently, meaning that patients received quick responses without having to wait for a physician’s consultation [35]. In addition, nurses reported that digital PROs helped patients be better prepared, leading to new topics being addressed in consultations, like fatigue and sexual dysfunction, supporting patient-centred care by focusing on issues worrying patients [34]. In this way, digital PRO assessments enhanced care quality by providing reliable patient responses and helped nurses decide when to consult a medical doctor [34]. Reflections were made on nurses should react to every reported symptom by the patients, where some nurses perceived a need to act on items reported in the digital PRO questionnaires, while others expressed doubt about whether to act on flagged questions, especially if only one question was flagged red [34].

- Ease of use

IBD nurses experienced the digital PRO system as user-friendly, and when technical issues appeared, technical support was available [34,36]. Likewise, nurses experienced that the system did not seem to be a problem for the patients either, but some patients struggled with website navigation and question comprehension due to dyslexia [34]. The CAPTURE-IBD intervention was seen as a user-friendly system due to its proactive care and telemonitoring features [36]. Initial concerns about increased workload and frequent patient interactions proved to be unfounded [37]. The system became easier to use after initial start-up, but it required some effort initially, e.g., loading information, but the payoff was accessible knowledge and effective communication [37]. Fear of increased workload initially hindered interdisciplinary usage, but those who engaged with the system recognized its value, indicating that its ease of use and effectiveness became apparent over time [37].

#### 3.3.2. Barriers

- Increased workload and task imbalance

Nurses reported that telehealth interventions increased their workload, as patient symptom reporting required additional follow-ups [36]. In addition, implementing AmbuFlex shifted the annual follow-up care from gastroenterologists to nurses [34], resulting in less time for nursing duties [34]. If the coordinator was not a clinician, nurses might feel overburdened, suggesting that an algorithm could better identify patients needing different levels of remote monitoring [36]. It was also suggested that the coordinator role should be replaced with a nursing position to provide more direct patient support [36].

Managing advice lines contributed to anxiety among nurses due to extended workload, as many calls lasted between five and 15 min, excluding the “invisible” pre- and post-call work [35]. Patients often reached out to nurses, who were more accessible, for issues that could be handled by non-nursing staff, like appointment scheduling and financial questions [35], calling for administrative support staff in IBD clinics, allowing nurses to focus on medical issues. Consequently, strategies to proactively address clinical workload issues were found necessary [36].

- The need for customized interventions

Nurses experienced that patients found the digital PRO questionnaire to be excessively lengthy and that not all questions were relevant to every patient [34]. In particular, patients undergoing biological treatment found the questions being distributed too frequently and perceived the process as primarily beneficial for the clinic rather than for themselves [34]. The PRO intervention was particularly advantageous for patients experiencing significant changes in symptoms and for individuals newly diagnosed with their condition [36]. Nurses experienced that there was a recognized need for clearer action plans guided by patient-reported outcomes, and to better capture day-to-day changes in patients with comorbid conditions [36].

- Technical issues and concerns regarding the security of the digital system

Nurses reported issues with the digital system following updates, and experienced that some patients had difficulties accessing the website for the digital solution [34]. A significant concern was data security, which could impede the adoption of the system, although reassurances from the government and IT departments stated that it was secure [37]. Additionally, the digital system was noted to lack integration with existing systems, and nurses experienced that this prevented patients from accessing real-time test results for blood and faecal calprotectin, as initially promised [37].

- Telehealth; a supplementary option or a standard procedure

Nurses’ attitudes varied on whether digital PROs should be standardized or offered as an optional tool to patients with IBD [34]. While some nurses viewed it as an optional resource, others believed it should become a standard practice. However, nurses reported that patient portals were less useful for acute IBD patients, who frequently visited the hospital and primarily utilized the system for appointment changes and general information, suggesting that the portal might be more beneficial for patients who did not require regular hospital visits [37].

- Concerns related to the patient–nurse relationship

Nurses had various opinions regarding the impact of digital PROs on the patient–nurse relationship [34]. Nurses appreciated that the comment field helped maintain a connection with patients, while others expressed concerns about not having met patients in person, viewing this as a potential drawback [34,35]. Some nurses noted that patients were sceptical about the new system, preferring in-person consultations both for a sense of security and because they valued in-person visits even when feeling well. Other nurses reassured patients that they were seen every three years and could call for support anytime [34].

## 4. Discussion

This scoping review mapping nurses’ experiences with telehealth in the follow-up care of patients with IBD identified three main benefits and five significant barriers, while also highlighting the similarities and differences among various studies regarding these experiences.

Similarities across studies highlight the significant role of digital PROs in empowering patients by improving patients’ health literacy, disease knowledge, and self-care skills, while regular symptom tracking fosters greater confidence in self-management. There is a shared recognition that telehealth solutions have increased accessibility to healthcare, especially for stable IBD patients. Nurses emphasize that the ability to provide proactive support through digital consultations enhances continuity of care, which is vital for managing chronic conditions like IBD. However, across the research, nurses express concerns about increased workload stemming from telehealth interventions. Specifically, managing patient queries and follow-ups related to symptom reporting can detract from traditional nursing responsibilities. While digital tools can enhance efficiency, they also introduce new complexities that require careful management. The studies reveal a divergence in nurses’ perceptions regarding the role of digital PROs in patient care. While some studies advocate for making digital PROs a standard part of treatment, others suggest they should be optional tools, reflecting varied perspectives among nurses about their applicability to different patient populations, particularly distinguishing between acute and stable cases. Moreover, technical and security challenges are identified as barriers to effective implementation of telehealth systems, with some studies focusing on data security concerns and others on the user-friendliness of the platforms. The impact of digital consultations on the patient–nurse relationship varies; some nurses value the relational elements maintained through digital PROs, while others worry about reduced in-person interaction, suggesting that the transition to digital care may not uniformly enhance or detract from established relationships.

### 4.1. Nurses’ Experiences on Benefits Conducting Telehealth

Our findings reveal that IBD nurses experience that telehealth facilitates prompt access to care and the management of patients’ disease progression, as well as empower patients in their self-care efforts and enhances their health literacy skills. The findings indicated that nurses perceived telehealth as an optimization of staffing time, which consequently facilitated improved two-way communication and allowed for greater focus on acute care. Additionally, nurses reported an increase in patient-centred care and the opportunity to address new topics during consultations. The use of digital PROs was characterized as user-friendly, and nurses noted that the ease of use improved over time.

Our findings indicate that nurses experience that telehealth provides patients with a better overview of their disease, which is imperative given the unpredictable course of IBD [5,6]. Health literacy plays a crucial role in enhancing patients’ understanding of their diseases and empowering them in their healthcare decisions [23,39]. Health literacy is defined as the skills patients use to stay healthy. Specifically, it encompasses skills to access, process, comprehend, and utilize health-related information effectively [39]. Similar impacts of telehealth have also been observed in studies concerning other chronic conditions [40,41]. Moreover, engaging patients in meaningful conversations with healthcare providers can significantly improve their healthcare experience, making patients feel respected, listened to, and empowered [42]. In addition, we found that telehealth enables patient-centred follow-up care and enhances care quality by providing reliable patient responses, supporting tailored care planning according to patients’ needs and thus helping nurses decide when to consult a medical doctor. The implementation of a “traffic light” scoring system helps nurses streamline care by identifying patients’ needs early and giving quick responses, often reducing the necessity for hospital visits or physician consultations. Furthermore, previously underreported issues within this patient group, such as fatigue and sexual dysfunction, are more likely to be discussed with nurses because patients are now better prepared to engage in these conversations.

Given the increased pressure on the healthcare system due to the increasing number of patients with chronic and complex diseases [15], as well as the increased attention on making patients active participants, we found that telehealth allows patients to contact nurses, enabling them to manage their own follow-up care because PROs facilitate increased insight into their symptom patterns. In this way, symptom monitoring can enhance patients’ self-care skills in managing disease-related symptoms through medication adherence, dietary modifications, physical activity, and stress management [36,43]. This aligns with Bandura’s social cognitive theory arguing that self-efficacy plays a crucial role in influencing individuals’ motivation and ability to engage in health-promoting behaviors, such as self-care [44]. Other studies have similarly reported enhancements in patients’ general understanding of disease-related knowledge, highlighting that telehealth facilitates continuous education [41]. This enables nurses to convey information regarding diagnoses, treatment plans, and preventive care in a manner that is more accessible to patients [41]. However, patients’ lack of knowledge regarding their condition becomes evident when recording PRO data [34], a factor that nurses should consider in their follow-up care. Limited health literacy can therefore cause significant challenges, and the manner in which health information is presented affects how patients perceive disease risks and ultimately influences their health-related behaviors [39].

Telehealth allows both nurses and patients to follow symptom trends over time, leading to earlier interventions in both physical and mental health [40]. Nurses in our study experienced that telehealth could benefit patients’ mental health, given that it often remains insufficiently integrated into IBD care. Nurses also experienced that newly diagnosed patients reported better navigation of their healthcare through telehealth due to enhancements towards more proactive disease management, reducing the need for physical consultations. Previous studies have also shown that digital follow-up care may enhance patients’ quality of life, medication adherence, and overall satisfaction compared to physical consultations in patients with IBD [5,21,41]. IBD nurses reported that the digital PRO system was user-friendly, with technical support available. Our findings are consistent with prior research in diabetes and palliative care, indicating that healthcare personnel generally perceive telehealth as user-friendly [17,45,46]. However, achieving digital empowerment among nurses necessitates access to information, support, and opportunities for professional development, as unstructured training has been associated with frustration and increased intentions to resign [47]. Nevertheless, this shift towards greater patient involvement highlights the importance of providing adequate training for IBD nurses to gain skills in digital communication and coaching strategies, triage, decision-making, and leadership, to be prepared to deliver telehealth services in accordance with the N-ECCO guidelines [12]. This also leads to the need to overcome barriers related to technical issues, as well as the necessity for effective methods for learning, training, and support [14]. The most influential factor for implementation of telehealth is technological competence, and studies have found that telehealth is only useful if providers are properly trained [45]. Education on telehealth should be implemented into nursing education, making nurses confident in their skills and knowledge about telehealth to be able to improve health outcomes in patients [12,13,48]. Such integration also has the potential to facilitate the dissemination of this knowledge to future colleagues in the professional environment [45]. This assertion is supported by the fact that most articles were published after the COVID-19 pandemic, reinforcing the theory of increased telehealth adoption during this period [12,13,20]. Although telehealth follow-ups for IBD patients began before the pandemic, the findings indicate a rising trend, which may result in an increased demand for nurses skilled in this area.

### 4.2. Nurses Experiences on Barriers Conducting Telehealth

Our findings indicate that nurses faced an increased workload due to additional follow-ups, task shifting, pre- and post-call activities, and more frequent patient outreach attributed to their greater accessibility. Nurses also expressed a need for tailored interventions and voiced concerns about data security and the lack of integration with existing systems. There were divergent opinions among nurses regarding whether telehealth should serve as a supplementary option or a standard procedure, as well as varied perspectives on the impact of digital PROs on the patient–nurse relationship. Some nurses expressed stress over handling increased volumes of digital information and struggled to integrate telehealth tasks into their existing workflow. Resistance from senior clinicians and limited organizational support were barriers to a broader implementation of telehealth. This aligns with previous findings, which indicate that successful adoption of digital innovations into routine care often requires both acceptance and motivation among health personnel [17]. It was suggested that assigning the coordinator role to IBD nurses and implementing algorithms to help identify patients who require closer monitoring would reduce nurses’ workload. Given that 61% of patients’ concerns were managed by nurses, including appointment bookings, insurance queries, and financial issues [35], there is a clear need for better sorting of incoming inquiries. Additionally, nurses spent considerable time on “invisible” tasks, such as preparation and follow-up related to patient contact, besides replacing traditional follow-up by gastroenterologists, resulting in nurses assuming greater responsibility for long-term monitoring. While this shift improved continuity for some patients, it reduced the time nurses could spend on their own follow-ups and increased their administrative burden. This is supported by studies among other patient groups, showing that telemedicine may involve more administrative work, may not always save clinicians time, and may lead to increased direct communication, such as e-mails [49]. Nurses also highlighted the extra time they spent on supporting patients with technical problems, noting that patients often contacted them when in need, as they were uncertain about whom to contact and found nurses easily accessible.

Although most nurses reported access to technical support, technical issues and concerns about data security were noted as barriers to implementation of telehealth, in line with various studies addressing uncertainty regarding patient privacy and access to patient data [17,40,45,50]. In addition, there was an initial expectation that the digital system would enhance information sharing among the multidisciplinary team and give patients easier access to their test results. However, nurses reported a lack of integration with the existing digital systems, affecting its usability.

While telehealth offers potential benefits, it is not a one-size-fits-all solution; it must cater to individual needs [40,45]. Nurses experienced that PRO questionnaires could be unsuitable for certain patients, particularly those receiving clinic-based treatment, citing issues like lengthy or irrelevant questions [34]. Nurses found digital follow-ups most beneficial for patients with significant symptom changes, especially newly diagnosed individuals [36]. A crucial aspect of patient-centred care is the patient’s engagement with their caregivers, which may enhance patients’ health outcomes, as shown through PROs [42]. Nurses emphasized the importance of a transparent, structured PRO-guided action plan to improve individualized follow-up care.

Telehealth has significantly influenced the patient–nurse relationship, presenting challenges for both parties [14,34]. Research shows that telehealth interactions differ from in-person encounters, affecting how nurses and patients communicate, particularly in terms of verbal and nonverbal cues [19]. The COVID-19 pandemic underscored these differences, particularly on how established versus new patient relationships were managed [22]. Building trust in virtual settings can be difficult, leading to possible communication barriers [49,50]. While some nurses in our studies appreciated the continuous contact telehealth provides, others worried about the absence of in-person interactions during telehealth consultations [34,35]. Likewise, nurses experienced that many patients expressed unease about losing in-person contact, valuing it even during periods of remission [34]. While asynchronous telehealth emphasizes flexibility and access, hybrid telehealth enhances patient engagement and continuity of care by leveraging the strengths of both modalities. Nurses held differing views, where some felt digital follow-up was better suited for patients with stable diseases or those rarely hospitalized, as well as serving as an alternative pathway for patients in deep remission [37]. Other studies view telehealth as a supplemental tool for illness management [46], which should be tailored to individual needs rather than replace in-person visits [40]. This patient-centred, holistic approach emphasizes viewing the patient as a whole individual, allowing them to be active participants in their care and follow-up plan [17]. Telehealth supports this by enhancing communication, support planning, and personalized treatment options, while enabling real-time symptom reporting that helps patients manage their illness effectively [51].

### 4.3. Strenghts and Limitations

One of the strengths of this review lies in our adherence to a recognized framework for scoping reviews, in conjunction with utilizing the PRISMA-ScR guidelines for reporting. We conducted a thorough and systematic search in collaboration with research librarians to identify relevant published studies. Additionally, the processes for study selection and data extraction were carried out independently by pairs of authors. However, this scoping review has several limitations. Despite systematic searches across multiple databases, we encountered a limited number of studies reporting IBD nurses’ experiences with telehealth, even after multiple updates, indicating a scarcity of research on this topic. However, not searching additional databases or including grey literature may have resulted in overlooking relevant publications. Additionally, by focusing exclusively on studies published in Scandinavian and English languages, we may have excluded important research in other languages, which could influence the international representation of our findings and introduce information bias. However, if including all languages, we would have to rely on AI-assisted translations, potentially compromising the quality of those translations. While the studies included are relevant and contribute to addressing the research question, it is important to note that critical appraisal is not typically part of scoping reviews, as they do not aim to assess the quality of evidence [26], differing from systematic reviews, which require quality appraisal of individual studies and risk-of-bias assessments [29]. However, the absence of quality assessment implies that the findings may be based on studies with varying methodological quality, even if peer reviewed. Furthermore, the included studies originate from Denmark, the US, Canada, and the UK, each characterized by unique cultural and health organizational contexts. Therefore, our findings should be interpreted with caution regarding their applicability to clinical practice.

### 4.4. Implications

The findings of this scoping review reveal notable benefits and barriers that may carry significant implications for nursing practice. This shift from a reactive to a proactive approach in follow-up will be an important part of patient care. Integrating this technology can lead to improved health outcomes and greater patient satisfaction. However, it is evident from the findings that there is a surprising lack of research on nurses’ experiences conducting telehealth for IBD patients, calling for further research in this innovative field of nursing practice. This gap hinders our understanding of how telehealth is implemented, perceived, and sustained in clinical practice. To fully realize the potential of telehealth, it is imperative to implement several practical strategies. Establishing triage protocols can effectively prioritize patient needs according to the severity of their conditions, facilitating tailored interventions. Moreover, the use of algorithmic alerts can promptly inform nurses of significant changes in patient-reported outcomes, thereby ensuring timely follow-up for pressing concerns. Integrating telehealth with electronic health records is vital for optimizing data accessibility during consultations, which enhances the overall quality of care. Additionally, comprehensive nurse training programs are essential to equip nurses with the digital competencies required for effective telehealth practice and to cultivate rapport with patients in virtual settings.

Future research should aim to explore and capture the depth and complexity of nurses’ experiences through qualitative methodologies. Furthermore, quantitative studies should assess telehealth models across diverse clinical settings and compare telehealth with traditional follow-up care and hybrid care. As additional primary studies emerge, systematic reviews will be essential for synthesizing evidence and facilitating quality appraisal. As IBD care evolves toward more integrated and digitally supported models, incorporating nurses’ perspectives in the development and evaluation of these services is essential to effectively meet the needs of both healthcare professionals and patients.

## 5. Conclusions

Nurses view telehealth as a promising approach that empowers patients’ health literacy and self-care skills and improves patient outcomes through effective monitoring. To optimize telehealth in IBD follow-up care, challenges such as increased workload, the necessity for tailored interventions, and technical and security issues must be addressed. Additionally, fully realizing telehealth’s potential requires implementing strategies like triage protocols to prioritize patient needs, algorithmic alerts for timely follow-up, integration with electronic health records for improved data accessibility, and comprehensive training programs for nurses to enhance digital competencies and foster patient rapport in virtual settings. Future research should investigate how telehealth can be embedded into nursing education, focusing on developing competencies related to digital health technologies and communication strategies. Studies comparing the impacts of synchronous, asynchronous, and hybrid methods on the patient–nurse relationship may provide valuable insights that further refine telehealth practices within nursing care.

## Figures and Tables

**Table 1:** The PCC framework showing descriptive elements in each category.

Population	Concept	Context
Inflammatory bowel disease	Perception	Web-based monitoring
IBD	View	Telehealth
Crohn Disease	Perspective	Telemedicine
Ulcerative Colitis	Experience	m-health/mhealth
	Attitude	Remote Consultation
	Evaluation	Remote patient consultation
	Thoughts	E-health/ehealth
	Reports	Digital health
	Satisfaction	Digital health monitoring
	Opinion	Digital home monitoring
	Feedback	Digital care
	Nurse–Patient Relations	Digital home care
	Nurses by role	Digital needs-based follow-up
	Nurse-led	
	Nurse-managed center	
	Nurse clinic	
	Nurse managed clinic	
	Nurse-delivered	
	Attitude of health personnel	

**Table 2:** Inclusion and exclusion criteria applied to the literature search.

Inclusion Criteria	Exclusion Criteria
Inflammatory bowel disease (Crohn’s disease and/or ulcerative colitis)	Other chronic diseases
Digital follow-up in the form of web-based platforms (asynchronous) as the only follow-up, or combined with either Synchronous real time telephone or video consultations or Physical consultation by a nurse	Standard consultation with physical consultation as the only follow-up
Nurses’ experiences	Patients’ experiences
IBD nurses (nurses and specialist nurses) alone, or as part of a team where data from nurses can be extracted	Data from doctors and other healthcare personnel
Follow-up of adults over 18 years of age	Follow-up of children
Full text available	Full text not available
English or Scandinavian languages	Languages other than English and Scandinavian
Peer-reviewed primary research articles with qualitative, quantitative, or mixed-method design	Conference abstracts, editorials, grey literature, theses, all types of reviews (systematic, scoping, integrative, umbrella and narrative)

**Table 3:** Specific search strategy in CINAHL.

CINAHL	Keywords/Index Terms/Phrases
Population	“(Mesh term) Inflammatory Bowel Disease” OR “Inflammatory bowel disease*” OR “IBD” OR “(Mesh term) Crohn Disease” OR “Crohn* disease” “(Mesh term) Colitis, Ulcerative” OR “Ulcerative colitis” OR “Crohn*” AND
Concept	“(Mesh term) Nurse-Patient Relations’ OR “Nurse-patient relation*” OR “(Mesh term) Nurses by Role+” OR “Nurs* role*” OR “Nurse-led*” OR “(Mesh term) Nurse-Managed Centers” OR “Nurse-Managed center*” OR “Nurs* clinic*” OR “Nurs* managed*” OR “(Mesh term) Nurse Attitudes” OR “Nurs* attitude*” OR “(Mesh term) Attitude of health personnel+” OR “Attitude* N3 (personnel or staff or professional*)” OR “(Mesh term) Perception+” OR “Perception*” OR “View*” OR “Perspective*” OR “Experience*” OR “(Mesh term) Attitude+” OR “Attitude*” OR “(Mesh term) Evaluation+” OR “Evaluation*” OR “Thought*” OR “(Mesh term) Reports+” OR “Report*” OR “Satisfaction” OR “Opinion*” OR “Feedback” OR “(Mesh term) Feedback” OR “Nurse delivered” AND
Context	“Web-based monitoring” OR “(Mesh term) Internet-based intervention” OR “Internet-based intervention” OR “(Mesh term) Telehealth+” OR “Telehealth” OR “(Mesh term) Telemedicine+” OR “Telemedicine” OR “Mhealth” OR “m-health” OR “(Mesh term) Remote Consultation” OR “remote consultation*” OR “remote patient monitoring” OR “ehealth” OR “e-health” OR “(Mesh term) Digital Health+” OR “Digital health*” OR “Digital Health monitoring” OR “Digital care*”

* “Exp” indicates that the term was exploded to include all narrower terms.

**Table 4:** Data extraction form showing selected publications.

First Author, Year, Country	Aim	Setting	Participants	Digital Platform	Context of Telehealth	Methods (Design, Data Collection Method, Data Analysis)	Data Collection Period	Key Findings: Benefits	Key Findings: Barriers	Conclusion
Amalie Søgaard Nielsen, 2022, Denmark. [34]	To explore hospital clinicians’ attitudes and rationales towards digital PROs used in the basic care of IBD.	Outpatient IBD clinic providing care to 850 patients with IBD.	Six RNs and six MDs.	Digital PRO platform called AmbuFlex IBD.	Hybrid. Asynchronous combined with in-person consultations or phone calls.	Action research, qualitative semi-structured face-to-face interviews, thematic analysis.	October 1st to December 20th, 2018.	Empower patients and boost their health literacy. Better prepared to consultations. Enhance two-way communication. Increase accessibility to healthcare. Timely monitoring. Improve quality of care due to more reliable real time data. More focus on issues worrying patients. Optimal use of staff by reducing consultation time with healthy patients. Ease of use.	Time consuming to manage and coordinate the digital PRO system. Redistributed care from MDs to RNs. Question overload in the PRO questionnaires. Technology challenges. Patient engagement variability. Clinicians’ attitudes. Patient–nurse relationship.	Digital PROs are useful tools to prioritise resources and prevent avoidable face-to-face consultations. Digital PRO-based follow up is recommended being initiated when a relationship is established.
Usha Chauhan, 2021, Canada. [35]	To capture utilization of phone and e-mail services among Canadian IBD nurses for 14 days.	Academic centres and community centres.	Twenty-one IBD nurses from 16 centres across Canada. 572 patients nurse encounters were reported.	E-mail communication.	Hybrid. Asynchronous and synchronous (telephone and email).	Cross-sectional, survey with 8 questions, statistical analyses.	Between 1 May 2017 and 30 June 2017.	Improved access to care and continuity of nursing care. Timely monitoring. Optimal use of staff time avoiding unnecessary clinic visits and calls to gastroenterologists. User-friendly.	Increased workload as RNs manage numerous calls that could have been handled by non-nursing staff, such as scheduling appointments, addressing insurance and financial concerns. Absence of in-person interaction.	Nurses were able to address 61% of IBD patients’ concerns independently, helping patients avoid contacting other health services.
Daniel Aintabi, 2024, USA. [36]	To evaluate the perspective on CAPTURE-IBD from a patient and clinician perspective, and perspective on care coordinator-triggered algorithms.	A gastro-enterology clinic at a tertiary referral centre.	Two nurses, two gastroenterologists, and the CAPTURE-IBD care coordinator.	Digital PRO platform called CAPTURE-IBD.	Asynchronous.	Qualitative, individual face-to-face interviews, rapid qualitative analysis.	Between April 2019 and January 2020.	Support symptom monitoring and psychosocial care. Support action plans between visits. User-friendly. Enhance patient engagement and improve communication.	Increase nursing workload. Should better capture day-to-day changes in patients with comorbid conditions. Need for clearer action plans guided by patient-reported outcomes.	Remote PRO monitoring is valuable, allowing for more frequent assessments in patients with active IBD and less frequent monitoring for those with inactive IBD. Increased workload issues need to be addressed in PRO-guided care pathways to be sustainable.
Elaine Bidmead, 2016, UK. [37]	To understand the barriers and benefits from using the PKB-PHR system and share the findings.	Gastro-enterology department of an English NHS foundation trust hospital.	Three IBD nurse specialists.	Patient-held personal health records (PHRs) called Patients Know Best (PKB).	Asynchronous.	Case study, semi-structured individual telephone interviews, thematic analysis.	Between January and February 2015.	Empower patients by increasing confidence and self-management. Enhance two-way communication. Improve access to care. Timely monitoring. Optimal use of staff time. Cost effective. Easier to use after initial start-up.	Technical challenges. Need for adequate training. Fear of more work. Clinicians attitude. Data security concerns. Problems with data integration. Variability in patient engagement.	The PKB system helps nurses in empowering patients by creating streamlined pathways for stable IBD patients to access information and proactive support, resulting in greater patient confidence, ownership of their condition, adherence to medication regimens, and enhanced self-management.

## Data Availability

No new data were created or analyzed in this study. Data sharing is not applicable to this article.

## Supplementary Materials

The following supporting information can be downloaded at: https://www.mdpi.com/article/10.3390/nursrep16010011/s1, Supplement S1: PRISMA-ScR checklist. Supplement S2: Keywords and index terms. Supplement S3: Search history from CINAHL. Supplement S4: Excluded articles with reasons.

## Public Involvement Statement

No public involvement in any aspect of this research.

## Guidelines and Standards Statement

This manuscript was prepared and reported in accordance with the Preferred Reporting Items for Systematic Reviews and Meta-Analyses extension for Scoping Reviews (PRISMA-ScR) Checklist [29].

## Abbreviations

The following abbreviations are used in this manuscript:

CD	Crohn’s Disease
JBI	Joanna Briggs Institute
IBD	Inflammatory Bowel Disease
MeSH	Medical Subject Headings
MD	Doctor of Medicine
PCC	Population, Concept, Context
PRESS	Peer Review of Electronic Search Strategies
PRISMA-ScR	Preferred Reporting Items for Systematic Reviews and Meta-Analyses
PROs	Patient-Reported Outcomes
RN	Registered Nurse
UC	Ulcerative Colitis

## References

[1] Yin A.L., Hachuel D., Pollak J.P., Scherl E.J., Estrin D. (2019). Digital Health Apps in the Clinical Care of Inflammatory Bowel Disease: Scoping Review. J. Med. Internet Res..

[2] Mak W.Y., Zhao M., Ng S.C., Burisch J. (2020). The epidemiology of inflammatory bowel disease: East meets west. J. Gastroenterol. Hepatol..

[3] Meier J., Sturm A., White L. (2019). Epidemiology. Inflammatory Bowel Disease Nursing Manual.

[4] Böhmig M., Sturm A., White L. (2019). Mechanisms of Disease, Etiology and Clinical Manifestations. Inflammatory Bowel Disease Nursing Manual.

[5] Fantini M.C., Loddo E., Di Petrillo A., Onali S. (2024). Telemedicine in inflammatory bowel disease from its origin to the post pandemic golden age: A narrative review. Dig. Liver Dis..

[6] Chauhan U., Sturm A., White L. (2019). Clinics. Inflammatory Bowel Disease Nursing Manual.

[7] Fiorino G., Allocca M., Chaparro M., Coenen S., Fidalgo C., Younge L., Gisbert J.P. (2018). ‘Quality of Care’ Standards in Inflammatory Bowel Disease: A Systematic Review. J. Crohn’s Colitis.

[8] Yu N., Wu K., Samyue T., Fry S., Stanley A., Ross A., Malcolm R., Connell W., Wright E., Ding N.S. (2024). Outcomes of a Comprehensive Specialist Inflammatory Bowel Disease Nursing Service. Inflamm. Bowel Dis..

[9] Al-Sheikh M., Ankersen D.V., Olsen J., Spanggaard M., Peters-Lehm C.T., Naimi R.M., Bennedsen M., Burisch J., Munkholm P. (2024). The Costs of Home Monitoring by Telemedicine vs. Standard Care for Inflammatory Bowel Diseases—A Danish Register-Based, 5-Year Follow-up Study. J. Crohn’s Colitis.

[10] Nguyen N.H., Martinez I., Atreja A., Sitapati A.M., Sandborn W.J., Ohno-Machado L., Singh S. (2022). Digital Health Technologies for Remote Monitoring and Management of Inflammatory Bowel Disease: A Systematic Review. Am. J. Gastroenterol..

[11] van Linschoten R.C.A., Visser E., Niehot C.D., van der Woude C.J., Hazelzet J.A., van Noord D., West R.L. (2021). Systematic review: Societal cost of illness of inflammatory bowel disease is increasing due to biologics and varies between continents. Aliment. Pharmacol. Ther..

[12] Charalambous J., Hollingdrake O., Currie J. (2024). Nurse practitioner led telehealth services: A scoping review. J. Clin. Nurs..

[13] Lerret S.M., Nuccio S., Compton A., Keegan M., Rapala K. (2023). Nurses’ Experiences and Perspectives of the Telehealth Working Environment and Educational Needs. J. Contin. Educ. Nurs..

[14] Koivunen M., Saranto K. (2018). Nursing professionals’ experiences of the facilitators and barriers to the use of telehealth applications: A systematic review of qualitative studies. Scand. J. Caring Sci..

[15] Fant C., Adelman D.S., Summer G.A. (2021). COVID-19 and telehealth: Issues facing healthcare in a pandemic. Nurse Pract..

[16] Roy J., Levy D.R., Senathirajah Y. (2022). Defining Telehealth for Research, Implementation, and Equity. J. Med. Internet Res..

[17] Lundereng E.D., Nes A.A.G., Holmen H., Winger A., Thygesen H., Jøranson N., Borge C.R., Dajani O., Mariussen K.L., Steindal S.A. (2023). Health Care Professionals’ Experiences and Perspectives on Using Telehealth for Home-based Palliative Care: Scoping Review. J. Med. Internet Res..

[18] Weldring T., Smith S.M.S. (2013). Patient-Reported Outcomes (Pros) and Patient-Reported Outcome Measures (Proms). Health Serv. Insights.

[19] Chen K., Huang J.J., Torous J. (2024). Hybrid care in mental health: A framework for understanding care, research, and future opportunities. NPP—Digit. Psychiatry Neurosci..

[20] Hakak F., Patel R.N., Gearry R.B. (2024). Review article: Telecare in gastroenterology—Within the COVID-19 pandemic and beyond. Aliment. Pharmacol. Ther..

[21] Aguas M., Del Hoyo J., Vicente R., Barreiro-de Acosta M., Melcarne L., Hernandez-Camba A., Madero L., Arroyo M.T., Sicilia B., Chaparro M. (2024). Telemonitoring of Active Inflammatory Bowel Disease Using the App TECCU: Short-Term Results of a Multicenter Trial of GETECCU. J. Med. Internet Res..

[22] Adelman D.S., Fant C., Summer G. (2021). COVID-19 and telehealth: Applying telehealth and telemedicine in a pandemic. Nurse Pract..

[23] Håkansson Eklund J., Holmström I.K., Kumlin T., Kaminsky E., Skoglund K., Höglander J., Sundler A.J., Condén E., Summer Meranius M. (2019). “Same same or different?” A review of reviews of person-centered and patient-centered care. Patient Educ. Couns..

[24] Oudbier S.J., Souget-Ruff S.P., Chen B.S.J., Ziesemer K.A., Meij H.J., Smets E.M.A. (2024). Implementation barriers and facilitators of remote monitoring, remote consultation and digital care platforms through the eyes of healthcare professionals: A review of reviews. BMJ Open.

[25] Gravina A.G., Pellegrino R., Durante T., Palladino G., D’Onofrio R., Mammone S., Arboretto G., Auletta S., Imperio G., Ventura A. (2023). Telemedicine in inflammatory bowel diseases: A new brick in the medicine of the future?. World J. Methodol..

[26] Peters M., Godfrey C., McInerney P., Munn Z., Tricco A., Khalil H., Aromataris E., Lockwood C., Porritt K., Pilla B., Jordan Z. (2024). Scoping Reviews. JBI Manual for Evidence Synthesis.

[27] Arksey H., O’Malley L. (2005). Scoping studies: Towards a methodological framework. Int. J. Soc. Res. Methodol..

[28] Levac D., Colquhoun H., O’Brien K.K. (2010). Scoping studies: Advancing the methodology. Implement. Sci..

[29] Tricco A.C., Lillie E., Zarin W., O’Brien K.K., Colquhoun H., Levac D., Moher D., Peters M.D.J., Horsley T., Weeks L. (2018). PRISMA Extension for Scoping Reviews (PRISMA-ScR): Checklist and Explanation. Ann. Intern. Med..

[30] Pollock D., Peters M.D.J., Khalil H., McInerney P., Alexander L., Tricco A.C., Evans C., de Moraes É.B., Godfrey C.M., Pieper D. (2023). Recommendations for the extraction, analysis, and presentation of results in scoping reviews. JBI Evid. Synth..

[31] McGowan J., Sampson M., Salzwedel D.M., Cogo E., Foerster V., Lefebvre C. (2016). PRESS Peer Review of Electronic Search Strategies: 2015 Guideline Statement. J. Clin. Epidemiol..

[32] Covidence. Reviewers. https://www.covidence.org/reviewers/.

[33] Ringnes H.K., Thørrisen M.M. (2024). Scoping Review—A Systematic and Flexible Method for Knowledge Synthesis (Scoping Review—En Systematisk og Fleksibel Metode for Kunnskapsoppsummering).

[34] Nielsen A.S., Appel C.W., Larsen B.F., Hanna L., Kayser L. (2022). Digital patient-reported outcomes in inflammatory bowel disease routine clinical practice: The clinician perspective. J. Patient-Rep. Outcomes.

[35] Chauhan U., Stitt L., Rohatinsky N., Watson M., Currie B., Westin L., McCaw W., Norton C., Nistor I. (2021). Patients’ Access to Telephone and E-mail Services Provided by IBD Nurses in Canada. J. Can. Assoc. Gastroenterol..

[36] Aintabi D., Greenberg G., Berinstein J.A., DeJonckheere M., Wray D., Sripada R.K., Saini S.D., Higgins P.D.R., Cohen-Mekelburg S. (2024). Remote Between Visit Monitoring in Inflammatory Bowel Disease Care: A Qualitative Study of CAPTURE-IBD Participants and Care Team Members. Crohn’s Colitis 360.

[37] Bidmead E., Marshall A. (2016). A case study of stakeholder perceptions of patient held records: The Patients Know Best (PKB) solution. Digit. Health.

[38] Aveyard H. (2023). Doing a Literature Review in Health and Social Care: A Practical Guide.

[39] Hasannejadasl H., Roumen C., Smit Y., Dekker A., Fijten R. (2022). Health Literacy and eHealth: Challenges and Strategies. JCO Clin. Cancer Inform..

[40] Dhunnoo P., Kemp B., McGuigan K., Meskó B., O’Rourke V., McCann M. (2024). Evaluation of Telemedicine Consultations Using Health Outcomes and User Attitudes and Experiences: Scoping Review. J. Med. Internet Res..

[41] Davis S.P., Ross M.S.H., Adatorwovor R., Wei H. (2021). Telehealth and mobile health interventions in adults with inflammatory bowel disease: A mixed-methods systematic review. Res. Nurs. Health.

[42] Santana M.J., Manalili K., Jolley R.J., Zelinsky S., Quan H., Lu M. (2018). How to practice person-centred care: A conceptual framework. Health Expect..

[43] Salmanpour N., Salehi A., Nemati S., Rahmanian M., Zakeri A., Drissi H.B., Shadzi M.R. (2025). The effect of self-care, self-efficacy, and health literacy on health-related quality of life in patients with hypertension: A cross-sectional study. BMC Public Health.

[44] Bandura A. (2004). Health Promotion by Social Cognitive Means. Health Educ. Behav..

[45] Medina Martin G., de Mingo Fernández E., Jiménez Herrera M. (2024). Nurses’ perspectives on ethical aspects of telemedicine. A scoping review. Nurs. Ethics.

[46] Wathne H., May C., Morken I.M., Storm M., Husebø A.M.L. (2024). Acceptability and usability of a nurse-assisted remote patient monitoring intervention for the post-hospital follow-up of patients with long-term illness: A qualitative study. Int. J. Nurs. Stud. Adv..

[47] Foong H.F., Kyaw B.M., Upton Z., Tudor Car L. (2020). Facilitators and barriers of using digital technology for the management of diabetic foot ulcers: A qualitative systematic review. Int. Wound J..

[48] Gartz J., O’Rourke J. (2021). Telehealth educational interventions in nurse practitioner education: An integrative literature review. J. Am. Assoc. Nurse Pract..

[49] Sloan M., Lever E., Harwood R., Gordon C., Wincup C., Blane M., Brimicombe J., Lanyon P., Howard P., Sutton S. (2021). Telemedicine in rheumatology: A mixed methods study exploring acceptability, preferences and experiences among patients and clinicians. Rheumatology.

[50] Alharbi A.R., Afaf Haroush Shaman A.-S., Modhi Hulayyil Salem A., Layla Sallam M., Al-Shabili A., Dalal Sayil A., Nemah Ahmed R., Modi N.A., Samirah Ali Ali M., Layla Abdullah A.-D. (2024). The Role of Nurse-Led Telehealth Interventions in Improving Healthcare Services and Patient Care. J. Int. Crisis Risk Commun. Res..

[51] Øvrebotten C.M., Hovland R.T., Låver J.C.Ø., Bentsen S.B., Moltu C. (2025). Healthcare professionals’ perceived challenges and benefits of digital patient-reported data for in-hospital postoperative pain monitoring: A qualitative study. Int. J. Nurs. Stud..

