# Supplementary material for: Nurses’ Experience Using Telehealth in the Follow-Up Care of Patients with Inflammatory Bowel Disease—A Scoping Review
Source: Nurs Rep. 2025 Dec 29;16(1):11. doi: 10.3390/nursrep16010011 (PMC12844754; doi:10.3390/nursrep16010011)
Supplement: Supplementary file 1 [file nursrep-16-00011-s001.zip › nursrep-4004893-supplementary.pdf]

SUPPLEMENT S1: PRISMA-SCR CHECKLIST

Preferred Reporting Items for Systematic reviews and Meta-Analyses extension for Scoping Reviews (PRISMA-ScR) Checklist

SECTION	ITEM	PRISMA-ScR CHECKLIST ITEM	REPORTED ON PAGE #
**TITLE**			
Title	1	Identify the report as a scoping review.	p. 1
**ABSTRACT**			
Structured summary	2	Provide a structured summary that includes (as applicable): background, objectives, eligibility criteria, sources of evidence, charting methods, results, and conclusions that relate to the review questions and objectives.	p. 1
**INTRODUCTION**			
Rationale	3	Describe the rationale for the review in the context of what is already known. Explain why the review questions/objectives lend themselves to a scoping review approach.	p. 2
Objectives	4	Provide an explicit statement of the questions and objectives being addressed with reference to their key elements (e.g., population or participants, concepts, and context) or other relevant key elements used to conceptualize the review questions and/or objectives.	p. 3
**METHODS**			
Protocol and registration	5	Indicate whether a review protocol exists; state if and where it can be accessed (e.g., a Web address); and if available, provide registration information, including the registration number.	p. 4
Eligibility criteria	6	Specify characteristics of the sources of evidence used as eligibility criteria (e.g., years considered, language, and publication status), and provide a rationale.	p.5
Information sources*	7	Describe all information sources in the search (e.g., databases with dates of coverage and contact with authors to identify additional sources), as well as the date the most recent search was executed.	p. 5
Search	8	Present the full electronic search strategy for at least 1 database, including any limits used, such that it could be repeated.	p. 6
Selection of sources of evidence†	9	State the process for selecting sources of evidence (i.e., screening and eligibility) included in the scoping review.	p. 6

SECTION	ITEM	PRISMA-ScR CHECKLIST ITEM	REPORTED ON PAGE #
Data charting process‡	10	Describe the methods of charting data from the included sources of evidence (e.g., calibrated forms or forms that have been tested by the team before their use, and whether data charting was done independently or in duplicate) and any processes for obtaining and confirming data from investigators.	p. 7
Data items	11	List and define all variables for which data were sought and any assumptions and simplifications made.	p. 6
Critical appraisal of individual sources of evidence§	12	If done, provide a rationale for conducting a critical appraisal of included sources of evidence; describe the methods used and how this information was used in any data synthesis (if appropriate).	NA
Synthesis of results	13	Describe the methods of handling and summarizing the data that were charted.	p. 7
**RESULTS**			
Selection of sources of evidence	14	Give numbers of sources of evidence screened, assessed for eligibility, and included in the review, with reasons for exclusions at each stage, ideally using a flow diagram.	p. 7
Characteristics of sources of evidence	15	For each source of evidence, present characteristics for which data were charted and provide the citations.	p. 8
Critical appraisal within sources of evidence	16	If done, present data on critical appraisal of included sources of evidence (see item 12).	
Results of individual sources of evidence	17	For each included source of evidence, present the relevant data that were charted that relate to the review questions and objectives.	p. 9
Synthesis of results	18	Summarize and/or present the charting results as they relate to the review questions and objectives.	p. 12
**DISCUSSION**			
Summary of evidence	19	Summarize the main results (including an overview of concepts, themes, and types of evidence available), link to the review questions and objectives, and consider the relevance to key groups.	p. 12
Limitations	20	Discuss the limitations of the scoping review process.	p. 18
Conclusions	21	Provide a general interpretation of the results with respect to the review questions and objectives, as well as potential implications and/or next steps.	p. 19
**FUNDING**			

SECTION	ITEM	PRISMA-ScR CHECKLIST ITEM	REPORTED ON PAGE #
Funding	22	Describe sources of funding for the included sources of evidence, as well as sources of funding for the scoping review. Describe the role of the funders of the scoping review.	p. 20

JBI = Joanna Briggs Institute; PRISMA-ScR = Preferred Reporting Items for Systematic reviews and Meta-Analyses extension for Scoping Reviews.
* Where *sources of evidence* (see second footnote) are compiled from, such as bibliographic databases, social media platforms, and Web sites.
† A more inclusive/heterogeneous term used to account for the different types of evidence or data sources (e.g., quantitative and/or qualitative research, expert opinion, and policy documents) that may be eligible in a scoping review as opposed to only studies. This is not to be confused with *information sources* (see first footnote).
‡ The frameworks by Arksey and O'Malley (6) and Levac and colleagues (7) and the JBI guidance (4, 5) refer to the process of data extraction in a scoping review as data charting.
§ The process of systematically examining research evidence to assess its validity, results, and relevance before using it to inform a decision. This term is used for items 12 and 19 instead of "risk of bias" (which is more applicable to systematic reviews of interventions) to include and acknowledge the various sources of evidence that may be used in a scoping review (e.g., quantitative and/or qualitative research, expert opinion, and policy document).

From: Tricco AC, Lillie E, Zarin W, O'Brien KK, Colquhoun H, Levac D, et al. PRISMA Extension for Scoping Reviews (PRISMAScR): Checklist and Explanation. Ann Intern Med. 2018;169:467–473. doi: 10.7326/M18-0850.

SUPPLEMENT S2 KEYWORDS AND INDEX TERMS

Table S1. Keywords and index terms used in all four electronic databases, with Boolean operators OR and AND*.

	**CINAHL**	**OVID MEDLINE AND EMBASE**	**WEB OF SCIENCE**
**Concept/context**	**Keywords/Index terms/phrases**	**Keywords/Index terms/phrases**	**Keywords/Index terms/phrases**
**Population**	"(Mesh term) Inflammatory Bowel Disease" OR "Inflammatory bowel disease*" OR "IBD" OR "(Mesh term) Crohn Disease" OR "Crohn* disease" "(Mesh term) Colitis, Ulcerative" OR "Ulcerative colitis" OR "Crohn*" AND	"Exp Inflammatory Bowel Diseases" OR "Inflammatory Bowel disease" OR "IBD" OR "Exp Crohn Disease" OR "Crohn* disease" OR "Exp Colitis, Ulcerative" OR "Ulcerative colitis" OR "Crohn*" AND	"Inflammatory bowel disease*" OR "IBD" OR "Crohn* disease*" OR "Ulcerative colitis" OR "Crohn*" AND
**Concept**	"(Mesh term) Nurse-Patient Relations' OR "Nurse-patient relation*" OR "(Mesh term) Nurses by Role+" OR "Nurs* role*" OR "Nurse-led*" OR "(Mesh term) Nurse-Managed Centers" OR "Nurse-Managed center*" OR "Nurs* clinic*" OR "Nurs* managed*" OR "(Mesh term) Nurse Attitudes" OR "Nurs* attitude*" OR "(Mesh term) Attitude of health personnel+" OR "Attitude* N3 (personnel or staff or professional*)" OR "(Mesh term) Perception+" OR "Perception*" OR "View*" OR "Perspective*" OR "Experience*" OR "(Mesh term) Attitude+" OR "Attitude*" OR "(Mesh term) Evaluation+" OR "Evaluation*" OR "Thought*" OR "(Mesh term) Reports+" OR	"Exp Nurse-Patient Relations" OR "Nurse-patient relation*" OR "Nurses by role" OR "Exp Nurse's Role" OR "Nurs* Role*" OR "Nurse-led*" OR "Nurse-managed centers" OR "Nurs* clinic" OR "Nurs* managed*" OR "Nurs* attitude*" OR "Exp Attitude of Health Personnel" OR "Attitude of health personnel" OR "Exp Perception" OR "Perception" OR "View" OR "Perspective" OR "Experience" OR "Exp Attitude" OR "Attitude" OR "Evaluation" OR "Though*" OR "Report*" OR "Satisfaction" OR "Opinion*" OR "Exp Feedback" OR "Feedback" OR "Nurse-delivered"	"Nurse-patient relation*" OR "nurs* role*" OR "nurse-led*" OR "nurse-managed center*" OR "nurs* clinic*" OR "nurs* managed*" OR "nurs* attitude*" OR "Attitude of health personnel" OR "perception*" OR "view*" OR "perspective*" OR "experience*" OR "attitude*" OR "evaluation*" OR "thought*" OR "report*" OR "satisfaction" OR "opinion*" OR "feedback" OR "nurse-delivered"

	"Report*" OR "Satisfaction" OR "Opinion*" OR "Feedback" OR ""(Mesh term) Feedback" OR "Nurse delivered" AND		
**Context**	"Web-based monitoring" OR "(Mesh term) Internet-based intervention" OR "Internet-based intervention" OR "(Mesh term) Telehealth+" OR "Telehealth" OR "(Mesh term) Telemedicine+" OR "Telemedicine" OR "Mhealth" OR "m-health" OR "(Mesh term) Remote Consultation" OR "remote consultation*" OR "remote patient monitoring" OR "ehealth" OR "e-health" OR "(Mesh term) Digital Health+" OR "Digital health*" OR "Digital Health monitoring" OR "Digital care*"	"Web-based monitoring" OR "Telehealth" OR "Exp Telemedicine" OR "Telemedicine" OR "mhealth" OR "m-health" OR "Exp Remote Consultation" OR "Remote consultation" OR "Remote monitoring" OR "Ehealth" OR "E-health" OR "Exp Digital Health" OR "Digital Health*" OR "Digital health monitoring" OR "Digital care*" OR "Digital follow-up"	"web-based monitoring" OR "Internet-based intervention" OR "telehealth" OR "telemedicine" OR "mhealth" OR "m-health" OR "remote consultation" OR "remote patient monitoring" OR "ehealth" OR "e-health" OR "digital health*" OR "digital health monitoring" OR "digital care*"

*"Exp" indicates that the term was exploded to include all narrower terms.

SUPPLEMENT S3 SEARCH HISTORY CINAHL

Table S2. Full search history from the CINAHL database, including search terms, Boolean operators, filters applied, and the number of results retrieved.

Search#	Query	Limiters/Expanders	Last Run Via	Results
S56	S7 AND S37 AND S55	Expanders - Apply equivalent subjects Search modes - Proximity	Interface - EBSCOhost Research Databases Search Screen - Advanced Search Database - CINAHL	157
S55	S38 OR S39 OR S40 OR S41 OR S42 OR S43 OR S44 OR S45 OR S46 OR S47 OR S48 OR S49 OR S50 OR S51 OR S52 OR S53 OR S54	Expanders - Apply equivalent subjects Search modes - Proximity	Interface - EBSCOhost Research Databases Search Screen - Advanced Search Database - CINAHL	77,884
S54	"internet-based intervention"	Expanders - Apply equivalent subjects Search modes - Proximity	Interface - EBSCOhost Research Databases Search Screen -	2,164

			Advanced Search Database - CINAHL	
S53	"digital care*"	Expanders - Apply equivalent subjects Search modes - Proximity	Interface - EBSCOhost Research Databases Search Screen - Advanced Search Database - CINAHL	140
S52	"digital health monitoring"	Expanders - Apply equivalent subjects Search modes - Proximity	Interface - EBSCOhost Research Databases Search Screen - Advanced Search Database - CINAHL	3
S51	"digital health*"	Expanders - Apply equivalent subjects Search modes - Proximity	Interface - EBSCOhost Research Databases Search Screen - Advanced Search Database - CINAHL	5,627
S50	(MH "Digital Health+")	Expanders - Apply equivalent subjects Search modes - Proximity	Interface - EBSCOhost Research Databases Search Screen - Advanced Search Database - CINAHL	33,353

S49	"e-health"	Expanders - Apply equivalent subjects Search modes - Proximity	Interface - EBSCOhost Research Databases Search Screen - Advanced Search Database - CINAHL	24,981
S48	"ehealth"	Expanders - Apply equivalent subjects Search modes - Proximity	Interface - EBSCOhost Research Databases Search Screen - Advanced Search Database - CINAHL	25,609
S47	"remote patient monitoring"	Expanders - Apply equivalent subjects Search modes - Proximity	Interface - EBSCOhost Research Databases Search Screen - Advanced Search Database - CINAHL	464
S46	"remote consultation*"	Expanders - Apply equivalent subjects Search modes - Proximity	Interface - EBSCOhost Research Databases Search Screen - Advanced Search Database - CINAHL	3,798
S45	(MH "Remote Consultation")	Expanders - Apply equivalent subjects	Interface - EBSCOhost	3,632

		Search modes - Proximity	Research Databases Search Screen - Advanced Search Database - CINAHL	
S44	"m-health"	Expanders - Apply equivalent subjects Search modes - Proximity	Interface - EBSCOhost Research Databases Search Screen - Advanced Search Database - CINAHL	23,902
S43	"mhealth"	Expanders - Apply equivalent subjects Search modes - Proximity	Interface - EBSCOhost Research Databases Search Screen - Advanced Search Database - CINAHL	25,167
S42	"telemedicine"	Expanders - Apply equivalent subjects Search modes - Proximity	Interface - EBSCOhost Research Databases Search Screen - Advanced Search Database - CINAHL	31,449
S41	"telehealth"	Expanders - Apply equivalent subjects Search modes - Proximity	Interface - EBSCOhost Research Databases Search Screen -	33,240

			Advanced Search Database - CINAHL	
S40	(MH "Telehealth+") OR (MH "Telemedicine+")	Expanders - Apply equivalent subjects Search modes - Proximity	Interface - EBSCOhost Research Databases Search Screen - Advanced Search Database - CINAHL	48,903
S39	(MH "Internet-Based Intervention")	Expanders - Apply equivalent subjects Search modes - Proximity	Interface - EBSCOhost Research Databases Search Screen - Advanced Search Database - CINAHL	1,950
S38	"web-based monitoring"	Expanders - Apply equivalent subjects Search modes - Proximity	Interface - EBSCOhost Research Databases Search Screen - Advanced Search Database - CINAHL	19
S37	S8 OR S9 OR S10 OR S11 OR S12 OR S13 OR S14 OR S15 OR S16 OR S17 OR S18 OR S19 OR	Expanders - Apply equivalent subjects Search modes - Proximity	Interface - EBSCOhost Research Databases Search Screen - Advanced Search Database - CINAHL	3,178,500

	S20 OR S21 OR S22 OR S23 OR S24 OR S25 OR S26 OR S27 OR S28 OR S29 OR S30 OR S31 OR S32 OR S33 OR S34 OR S35 OR S36			
S36	"nurse delivered"	Expanders - Apply equivalent subjects Search modes - Proximity	Interface - EBSCOhost Research Databases Search Screen - Advanced Search Database - CINAHL	374
S35	"feedback"	Expanders - Apply equivalent subjects Search modes - Proximity	Interface - EBSCOhost Research Databases Search Screen - Advanced Search Database - CINAHL	57,295
S34	(MH "Feedback")	Expanders - Apply equivalent subjects Search modes - Proximity	Interface - EBSCOhost Research Databases Search Screen -	22,444

			Advanced Search Database - CINAHL	
S33	"opinion*"	Expanders - Apply equivalent subjects Search modes - Proximity	Interface - EBSCOhost Research Databases Search Screen - Advanced Search Database - CINAHL	52,901
S32	"satisfaction"	Expanders - Apply equivalent subjects Search modes - Proximity	Interface - EBSCOhost Research Databases Search Screen - Advanced Search Database - CINAHL	172,314
S31	"report*"	Expanders - Apply equivalent subjects Search modes - Proximity	Interface - EBSCOhost Research Databases Search Screen - Advanced Search Database - CINAHL	1,073,941
S30	(MH "Reports+")	Expanders - Apply equivalent subjects Search modes - Proximity	Interface - EBSCOhost Research Databases Search Screen - Advanced Search Database - CINAHL	19,002

S29	"thought*"	Expanders - Apply equivalent subjects Search modes - Proximity	Interface - EBSCOhost Research Databases Search Screen - Advanced Search Database - CINAHL	64,737
S28	"evaluation*"	Expanders - Apply equivalent subjects Search modes - Proximity	Interface - EBSCOhost Research Databases Search Screen - Advanced Search Database - CINAHL	1,247,948
S27	(MH "Evaluation+")	Expanders - Apply equivalent subjects Search modes - Proximity	Interface - EBSCOhost Research Databases Search Screen - Advanced Search Database - CINAHL	78,723
S26	"attitude*"	Expanders - Apply equivalent subjects Search modes - Proximity	Interface - EBSCOhost Research Databases Search Screen - Advanced Search Database - CINAHL	454,438
S25	(MH "Attitude+")	Expanders - Apply equivalent subjects	Interface - EBSCOhost	590,163

		Search modes - Proximity	Research Databases Search Screen - Advanced Search Database - CINAHL	
S24	"experience*"	Expanders - Apply equivalent subjects Search modes - Proximity	Interface - EBSCOhost Research Databases Search Screen - Advanced Search Database - CINAHL	600,851
S23	"perspective*"	Expanders - Apply equivalent subjects Search modes - Proximity	Interface - EBSCOhost Research Databases Search Screen - Advanced Search Database - CINAHL	189,002
S22	"view*"	Expanders - Apply equivalent subjects Search modes - Proximity	Interface - EBSCOhost Research Databases Search Screen - Advanced Search Database - CINAHL	148,952
S21	"perception*"	Expanders - Apply equivalent subjects Search modes - Proximity	Interface - EBSCOhost Research Databases Search Screen -	207,034

			Advanced Search Database - CINAHL	
S20	(MH "Perception+")	Expanders - Apply equivalent subjects Search modes - Proximity	Interface - EBSCOhost Research Databases Search Screen - Advanced Search Database - CINAHL	96,843
S19	"attitude* N3 (personnel or staff or professional*)"	Expanders - Apply equivalent subjects Search modes - Proximity	Interface - EBSCOhost Research Databases Search Screen - Advanced Search Database - CINAHL	81,158
S18	"nurs* attitude*"	Expanders - Apply equivalent subjects Search modes - Proximity	Interface - EBSCOhost Research Databases Search Screen - Advanced Search Database - CINAHL	44,463
S17	(MH "Attitude of Health Personnel+")	Expanders - Apply equivalent subjects Search modes - Proximity	Interface - EBSCOhost Research Databases Search Screen - Advanced Search Database - CINAHL	140,039

S16	"nurs* managed*"	Expanders - Apply equivalent subjects Search modes - Proximity	Interface - EBSCOhost Research Databases Search Screen - Advanced Search Database - CINAHL	2,934
S15	"nurs* clinic*"	Expanders - Apply equivalent subjects Search modes - Proximity	Interface - EBSCOhost Research Databases Search Screen - Advanced Search Database - CINAHL	2,955
S14	"nurse-managed center*"	Expanders - Apply equivalent subjects Search modes - Proximity	Interface - EBSCOhost Research Databases Search Screen - Advanced Search Database - CINAHL	2,594
S13	(MH "Nurse-Managed Centers")	Expanders - Apply equivalent subjects Search modes - Proximity	Interface - EBSCOhost Research Databases Search Screen - Advanced Search Database - CINAHL	2,568
S12	"nurse-led*"	Expanders - Apply equivalent subjects	Interface - EBSCOhost	6,111

		Search modes - Proximity	Research Databases Search Screen - Advanced Search Database - CINAHL	
S11	"nurs* role*"	Expanders - Apply equivalent subjects Search modes - Proximity	Interface - EBSCOhost Research Databases Search Screen - Advanced Search Database - CINAHL	70,074
S10	(MH "Nurses by Role+")	Expanders - Apply equivalent subjects Search modes - Proximity	Interface - EBSCOhost Research Databases Search Screen - Advanced Search Database - CINAHL	177,004
S9	"nurse-patient relation*"	Expanders - Apply equivalent subjects Search modes - Proximity	Interface - EBSCOhost Research Databases Search Screen - Advanced Search Database - CINAHL	30,364
S8	(MH "Nurse-Patient Relations")	Expanders - Apply equivalent subjects Search modes - Proximity	Interface - EBSCOhost Research Databases Search Screen -	30,054

			Advanced Search Database - CINAHL	
S7	S1 OR S2 OR S3 OR S4 OR S5 OR S6	Expanders - Apply equivalent subjects Search modes - Proximity	Interface - EBSCOhost Research Databases Search Screen - Advanced Search Database - CINAHL	31,030
S6	"Ulcerative colitis"	Expanders - Apply equivalent subjects Search modes - Proximity	Interface - EBSCOhost Research Databases Search Screen - Advanced Search Database - CINAHL	8,537
S5	"crohn*"	Expanders - Apply equivalent subjects Search modes - Proximity	Interface - EBSCOhost Research Databases Search Screen - Advanced Search Database - CINAHL	12,193
S4	"crohn* disease*"	Expanders - Apply equivalent subjects Search modes - Proximity	Interface - EBSCOhost Research Databases Search Screen - Advanced Search Database - CINAHL	11,826

S3	(MH "Crohn Disease") OR (MH "Inflammatory Bowel Diseases") OR (MH "Colitis, Ulcerative")	Expanders - Apply equivalent subjects Search modes - Proximity	Interface - EBSCOhost Research Databases Search Screen - Advanced Search Database - CINAHL	20,268
S2	"IBD"	Expanders - Apply equivalent subjects Search modes - Proximity	Interface - EBSCOhost Research Databases Search Screen - Advanced Search Database - CINAHL	13,842
S1	"Inflammatory bowel disease*"	Expanders - Apply equivalent subjects Search modes - Proximity	Interface - EBSCOhost Research Databases Search Screen - Advanced Search Database - CINAHL	15,486

SUPPLEMENT S4 EXCLUDED ARTICLES FROM DATABASE SEARCH, INCLUDED REASONS

NO	FIRST AUTHOR AND YEAR	TITLE	REASON FOR EXCLUSION
1	Avery et al. (2024)	Flexibility & Accessibility, e-Literacy, Resourcing and The Human Factor: Early Lessons from EIBD, a UK Qualitative Interview Study	Conference abstract
2	Bouri et al. (2021)	What is the patient's and multidisciplinary team's perspective on telephone clinics?	Wrong intervention
3	Con et al. (2018)	Design considerations for an eHealth decision support tool in inflammatory bowel disease self-management.	Wrong intervention
4	Krishnaprasad et al. (2020)	Crohn's Colitis Care (CCCare): bespoke cloud-based clinical management software for inflammatory bowel disease	Not nurses experience
5	Degens (2014)	Results from a feasibility study with the telemedicine tool myIBDcoach in the Netherlands	Conference abstract
6	De Jong et al. (2017)	Development and Feasibility Study of a Telemedicine Tool for All Patients with IBD: MyIBDcoach	Not nurses experience
7	Harris et al. (2020)	Evolution of an inflammatory bowel disease helpline and implications for service design and development.	Wrong intervention
8	Hubbard et al. (2023)	Young persons and healthcare professionals experience of virtual	Not nurses experience

		gastroenterology consultations: A multicentre survey conducted during the COVID-19 pandemic	
9	Karimi et al. (2023)	Health Communication Research Informs Inflammatory Bowel Disease Practice and Research: A Narrative Review	Not nurses experience
10	Karimi et al. (2021)	The effect of a nurse-led advice line and virtual clinic on inflammatory bowel disease service delivery: an Australian study	Not nurses experience
11	Kati et al. (2018)	Essential Assessment Templates for IBD RNs	Conference abstract
12	Kelso et al. (2018)	Can Smartphones Help Deliver Smarter Care for Patients With Inflammatory Bowel Disease?	Not nurses experience
13	Kim et al. (2021)	Development and feasibility of a web-based decision aid for patients with ulcerative colitis: Qualitative pilot study	Not nurses experience
14	Krishnaprasad (2018)	Crohn's colitis care (CCCare): Development and testing of a bespoke cloud-based clinical management system for inflammatory bowel disease (IBD)	Conference abstract
15	Krishnaprasad et al. (2017)	Crohn's Colitis Care (CCCare): A bespoke cloud-based clinical management system for inflammatory bowel disease-development and beta testing	Conference abstract
16	Lalanza et al. (2023)	Patient and Healthcare Professional Insights of Home- and Remote-Based	Not nurses experience

		Clinical Assessment: A Qualitative Study from Spain and Brazil to Determine Implications for Clinical Trials and Current Practice	
17	Maurud et al. (2024)	Exploring the foundations of a digital health information service for patients with inflammatory bowel disease: a mixed method study in Gravitate-Health	Wrong intervention
18	O'Connor (2011)	Criteria for success using an inflammatory bowel disease nurse telephone service.	Wrong intervention
19	Peek-Kuijt et al. (2018)	Self-management in inflammatory bowel disease: A perspective of patients and caretakers	Conference abstract
20	Rohatinsky et al. (2021)	Perspectives of health care use and access to care for individuals living with inflammatory bowel disease in rural Canada.	Wrong setting
21	Rohatinsky et al. (2024)	Exploring the Experiences of Virtual Inflammatory Bowel Disease Care in Saskatchewan.	Wrong intervention
22	Spartz et al. (2023)	Advances in Mobile Health for Inflammatory Bowel Disease	Not nurses experience
23	Squires et al. (2023)	Digital Technology to Improve Consultation in IBD Care: A Literature Review.	Not nurses experience
24	Van Citters et al. (2020)	Same-page care is associated with improved shared decision-making for patients with inflammatory bowel disease	Conference abstract
